# Low plasma haptoglobin is a risk factor for life-threatening childhood severe malarial anemia and not an exclusive consequence of hemolysis

**DOI:** 10.1038/s41598-018-35944-w

**Published:** 2018-12-03

**Authors:** Samuel Eneọjọ Abah, Florence Burté, Sandrine Marquet, Biobele J. Brown, Francis Akinkunmi, Gbeminiyi Oyinloye, Nathaniel K. Afolabi, Samuel Omokhodion, Ikeoluwa Lagunju, Wuraola A. Shokunbi, Mats Wahlgren, Hélia Dessein, Laurent Argiro, Alain J. Dessein, Boris Noyvert, Lilian Hunt, Greg Elgar, Olugbemiro Sodeinde, Anthony A. Holder, Delmiro Fernandez-Reyes

**Affiliations:** 1 Francis Crick Institute, 1 Midland Road, London, NW1 1AT United Kingdom; 2 Aix-Marseille University, Inserm GIMP, Labex ParaFrap, Marseille, 13385 France; 3 Aix-Marseille University, Inserm Laboratoire TAGC/U1090, Marseille, 13288 France; 4 Department of Paediatrics, College of Medicine, University of Ibadan, University College Hospital, Ibadan, Nigeria; 5 Childhood Malaria Research Group, College of Medicine, University of Ibadan, University College Hospital, Ibadan, Nigeria; 6 Department of Haematology, College of Medicine, University of Ibadan, University College Hospital, Ibadan, Nigeria; 7 Department of Microbiology, Tumour and Cell Biology, Karolinska Institutet, Stockholm, Sweden; 8 Department of Computer Science, Faculty of Engineering, University College London, Gower Street, London, WC1E 6BT United Kingdom

## Abstract

Severe Malarial Anemia (SMA), a life-threatening childhood *Plasmodium falciparum* malaria syndrome requiring urgent blood transfusion, exhibits inflammatory and hemolytic pathology. Differentiating between hypo-haptoglobinemia due to hemolysis or that of genetic origin is key to understand SMA pathogenesis. We hypothesized that while malaria-induced hypo-haptoglobinemia should reverse at recovery, that of genetic etiology should not. We carried-out a case-control study of children living under hyper-endemic holoendemic malaria burden in the sub-Saharan metropolis of Ibadan, Nigeria. We show that hypo-haptoglobinemia is a risk factor for childhood SMA and not solely due to intravascular hemolysis from underlying schizogony. In children presenting with SMA, hypo-haptoglobinemia remains through convalescence to recovery suggesting a genetic cause. We identified a haptoglobin gene variant, rs12162087 (g.-1203G > A, frequency = 0.67), to be associated with plasma haptoglobin levels (p = 8.5 × 10^−6^). The Homo-Var:(AA) is associated with high plasma haptoglobin while the reference Homo-Ref:(GG) is associated with hypo-haptoglobinemia (p = 2.3 × 10^−6^). The variant is associated with SMA, with the most support for a risk effect for Homo-Ref genotype. Our insights on regulatory haptoglobin genotypes and hypo-haptoglobinemia suggest that haptoglobin screening could be part of risk-assessment algorithms to prevent rapid disease progression towards SMA in regions with no-access to urgent blood transfusion where SMA accounts for high childhood mortality rates.

## Introduction

The annual global incidence of clinical malaria is estimated at about 216 million cases leading to approximately 445,000 deaths in 2016^1^, a slight increase in morbidity over what was previously reported^1,2^. The mortality rate of malaria was estimated to be 90% in Africa, and 80% of these deaths occur in sub-Saharan Africa and mostly in the under five years–of-age group due to severe, and often-overlapping syndromes such as Severe Malarial Anemia (SMA) and Cerebral Malaria (CM)^1,2^. A quarter of the global cases and a third of malaria-attributable childhood deaths occur in Nigeria, the most populous country of Africa^3–5^.

Malaria is characterized by increased inflammation and intravascular hemolysis due to schizogony leading to the release of free Hemoglobin (fHb) into the plasma^6–8^. Heme, the molecule bearing the iron moiety of fHb, can be oxidized by nitric oxide (NO) leading to oxidative stress-mediated damage through mediators such as lipid and hydrogen peroxides^9,10^. To avoid this, fHb is scavenged by plasma Haptoglobin (HP)^10,11^ forming a complex, thereby preventing fHb to be oxidized and potentially preventing further heme-mediated nitric oxide damage, renal excretion of iron and further oxidative damage^10–12^.

The *HP* gene is comprised of two co-dominant alleles, *HP1* and *HP2*, located on the long arm of chromosome 16^12–15^. The *HP1* allele has five exons and the *HP2* allele has seven exons (Supplementary Fig. 1). The *HP2* allele contains a 1.7 Kb intragenic duplication. HP is synthesized mainly by hepatocytes in response to increased levels of interleukins such as IL-6 and IL-1 and Tumor Necrosis Factor (TNF-α) during an acute inflammatory response^11,16^. The HP-fHb complex, formed by the binding of HP to fHb, is removed from circulation by CD163 receptors expressed on the surface of macrophages followed by internalization, where fHb is degraded and heme catabolized by heme oxygenase-1^17^.

A high level of fHb during severe hemolysis has been reported to be responsible for low HP plasma levels^18,19^ and therefore the circulatory level of HP has been used as a surrogate marker of intravascular hemolysis^18,20–23^. Similarly, Lactate Dehydrogenase (LDH) is known to be abundant in erythrocytes and its plasma level is elevated in the event of erythrocyte lysis. An elevated plasma level of LDH is also a surrogate marker of hemolysis in vascular pathophysiology^24–27^.

In some individuals, a complete lack of HP (ahaptoglobinemia) or low circulating HP (hypo-haptoglobinemia) can be observed^28^. Circulatory HP level shows ethnic and geographic variation with a high prevalence of hypo-haptoglobinemia in Africans compared with Europeans. Recent studies report that the average frequency of hypo-haptoglobinemia in Africa might be as high as 40%^18,29^ although with different and unknown etiologies^18,20,30–32^.

Studies on hypo-haptoglobinemia have provided conflicting views regarding its etiology in childhood malaria. On one hand, the low level of plasma HP found in malaria infections has been proposed to have a genetic basis^18,31,33–35^, while on the other hand the low level has been attributed to the malaria infection causing intravascular hemolysis^18,28,36^ and other immune pathological processes^37^. HP levels have been reported to recover in 75% in a study population following anti-malarial treatment^38^. While malaria infection induced hypo-haptoglobinemia should be reversed with anti-malarial treatment^18,20,30–32^, that of genetic etiology should not. Differentiating between these two etiological scenarios should provide insights into the role of HP in the establishment of mild or severe malaria syndromes.

We hypothesized that if low HP levels are exclusively a consequence of malaria infection they would return to normal on patients’ recovery. To test this hypothesis, we carried out a prospective case-control study of children (with well-defined follow-up through convalescence to recovery) living in the sub-Saharan 3-million inhabitants densely-populated urban metropolis of Ibadan, Nigeria, that is under hyper-endemic and holoendemic malaria burden. We assayed for levels of plasma HP and the markers of intravascular hemolysis fHb and LDH from admission through convalescence to recovery. Furthermore, we performed high throughput amplicon-based sequencing of the *HP* genes to identify genotypes associated with both circulatory HP levels and the malarial disease syndromes studied.

Here we show, by longitudinal follow-up of malaria-positive pediatric cohorts, that hypo-haptoglobinemia is a risk factor for acute onset childhood SMA, rather than the unique consequence of this syndrome, and it is likely of genetic etiology. We report for the first time a variant in the distal upstream region of the *HP* gene rs12162087 to be associated with circulatory HP level. We provide evidence that the variant is also associated with SMA.

## Materials and Methods

### Ethical Statement

The internationally recognized ethical committee at the Institute for Advanced Medical Research and Training (IMRAT) of the College of Medicine, University of Ibadan (COMUI) approved this research on the platform of the Childhood Malaria Research Group (CMRG) within the academic Department of Paediatrics, University College Hospital (UCH), as well as at school and Primary Care centers throughout the city of Ibadan with permit number: UI/EC/10/0130.

### Ethics, Consent and Permissions

Parents and/or guardians of study participants gave informed written consent in accordance with the World Medical Association ethical principles for research involving human subjects.

### Study Site

All study participants were recruited under the auspices of the CMRG at the 800-bed tertiary hospital, UCH in the city of Ibadan, Nigeria, in west sub-Saharan Africa. UCH Ibadan academic prestige, quality of care, costs and excellent accessibility from any point in the large metropolis makes it the preferred choice for all socio-economic sectors of the population. UCH Ibadan provides tertiary, secondary and it is associated with primary care centers across the city.

Ibadan is the second most densely populated urban metropolis in Nigeria with nearly 3 Million inhabitants. The city has a lengthy 8-month rainy season, with the average of 10 rainy days per month between May and October. Malaria transmission and severe disease occur throughout the year. Although severe malaria syndromes are predominant in children under 5 years-of-age, there is a significant large burden of severe disease in children up to 16 years-of-age^39^.

### Study Design and Case Definitions

As part of a larger case-control study of severe malaria^4,5,39,40^, we recruited children with median age of 26–48 months-of-age between 2010 and 2013. The defined clinical cases for the malaria-positive groups include Uncomplicated Malaria (UM), Severe Malaria Anemia (SMA) and Cerebral Malaria (CM) according to WHO criteria for diagnosis of *P. falciparum* complications^41,42^. UM cases were defined as patients with fever and *P. falciparum* parasitemia who did not require hospital admission. The SMA group was defined as conscious children with packed cell volume (PCV) <16%, or hemoglobin concentration <5 g/dL, with asexual forms of *P. falciparum* in circulation and no other overt etiology. All SMA patients were transfused within two hours of admission and confirmation of malaria diagnosis. However, samples were collected prior to the blood transfusion. CM cases were defined as children with unarousable coma persisting for more than an hour with generalized convulsion in the presence of asexual forms of *P. falciparum*. A Blantyre coma score <2 was used to define coma status. Children presenting with both CM and SMA, belonging to the CM-SMA clinical group, did not form part of this study.

The UM group was recruited as part of daily routine malaria parasite screening at our children outpatient clinics at UCH Ibadan. All severe CM and SMA cases were recruited on admission from our emergency department at UCH Ibadan, the Otunba Tunwase Children’s Emergency Ward (OTCHEW). The Community Control (CC) group was comprised of malaria-negative healthy children.

All children diagnosed with malaria were treated and followed through convalescence to recovery following Nigerian antimalarial guidelines which are based on the WHO guidelines^43^. Uncomplicated malaria is treated with a 3-day oral course of Artemisinin-based Combination Therapy (ACT). On the other hand, severe malaria is treated with intravenous artesunate for at least 24 hours and until they can tolerate oral therapy after which treatment is then completed with three days of oral ACT. Overall, successful antimalarial treatment is completed in less than one week. At our clinics at UCH Ibadan, an urban academic tertiary-care center, guidelines are strictly followed and monitored weekly.

All cohorts excluded children presenting with: moderate to severe malnutrition and/or iron deficiency; hypochromic microcytic anemia; spherocytosis; sickle cell anemia (SS); haemoglobin (SC) disease; known beta-thalassemia status. Only the alpha-thalassemia –α^3.7^ deletion, which is of little or no clinical significance, is observed in Nigerians^44^. Beta-thalassemia trait is known to have a very low gene frequency of 0.008 in this population^45^ and recent authors report inconclusive higher prevalence within healthy individuals^46^.

The study was prospectively performed on subjects recruited from 2010 to 2012 (Discovery Cohort) and 2013 (Validation Cohort). The entire cohort is predominantly of the same Yoruba ethnicity and within the same population geopolitical zone.

All clinical malaria-positive groups received anti-malaria treatment. A child was considered recovered if, and only if, the PCV and reticulocyte counts returned to normal ranges; there were no clinical nor laboratory signs of hemolysis with fHb and LDH within normal range and; with a negative malaria Giemsa blood-film at day 28–35, day 28 (PD28) on the average. All these are well-defined clinical and physiological signs that a child has recovered from the acute malaria episode and are consistent with previous studies showing that Hb, reticulocyte counts and haptoglobin levels return to normal 2–3 weeks after commencement of antimalarial treatment^31,47–50^. As shown by these studies and our data, the PD28 recovery time-point is sufficient for the HP level and other clinical parameters to normalize as it happens in the CM and UM clinical groups.

### Clinical Data and Sample Collection

Clinical data were collected using a malaria-tailored questionnaire designed by the CMRG^4,5,39,40^ at University College Hospital Ibadan. The experiments were conducted using samples from 3 well-defined cohorts namely the discovery, the validation and the High-throughput Sequencing (HTS) cohorts.

For the discovery cohort, plasma samples were collected at acute onset of the disease termed as PD0 (Plasma at Day zero) as well as from parasite-negative healthy community controls; CC.

For the validation cohort plasma samples were collected at acute onset of the disease at day zero (PD0) during convalescence at day 7 (PD7) and day 14 (PD14); and at recovery at day 28–32 (PD28).

The HTS cohort is drawn from both the discovery and validation cohorts, including malaria-negative healthy controls, whose *HP* genes were sequenced.

All plasma samples from malaria-positive clinical groups within all these cohorts were collected prior to onset of anti-malarial drug treatment or blood transfusion in the case of SMA.

A 2.5 ml blood sample was obtained from each participant. A very short needle was used to collect the blood into a tube, which was firmly screwed to the syringe. This was to avoid dispensing blood into EDTA tubes through the syringe, which might have caused hemolysis of some red blood cells. Blood samples in an EDTA collection tube were kept on ice and plasma for each subject was harvested following centrifugation. Plasma for this study was harvested by centrifugation (1000 *g*, 10 minutes), aliquoted and stored at −80 °C within 2 hours of collection.

PCV was measured using micro-hematocrit capillary tubes centrifuged at 12,000 *g* for 5 min. The cell volume was calculated as a percentage of the whole tube volume.

### Malaria Diagnosis

Malaria parasites (MPs) were detected and counted by microscopy following Giemsa staining of thick and thin blood films. The criterion for declaring a participant to be malaria parasite-free was no detectable parasites in 100 high-power (1000x) fields in both thick and thin films. We validated the diagnosis outcome by randomly selecting one in ten thick blood films for independent review by local experienced senior microscopists who were not part of the study research team^4,5,39,40^.

### Quantitative Determination of Plasma Haptoglobin

HP levels in plasma were quantified using an HP sandwich enzyme-linked immunosorbent assay (sELISA) kit (Genway). Plasma samples diluted 1 in 20 in phosphate buffered saline (PBS) as stock were further diluted to 1/50, 1/100, 1/250, 1/5,000, 1/10,000, 1/20,000 and 1/40,000. The low to high dilutions were to ensure detection of HP that could be present at low to high concentration, respectively. Standards were prepared according to the manufacturer’s instructions but diluted in PBS. Anti-human HP antibody (Genway) was diluted in coating buffer (0.05M sodium carbonate-bicarbonate, pH 9.6) to 5 µg/mL. Plates were incubated with this antibody for 60 minutes at room temperature. Thereafter, a blocking step was performed with 400 μL of blocking buffer (50 mM Tris-HCl, 140 mM NaCl, 1% BSA, pH 8.0) followed by a washing step in washing buffer (0.05% Tween 20 in PBS, pH 7.4). Diluted plasma samples were added to the plates and incubated for 60 minutes, followed by another washing step. Bound HP was detected with a secondary antibody coupled with horseradish peroxidase, which was diluted 1 in 2,000 in 50 mM Tris-HCl containing 140 mM NaCl, 1% BSA (wt/vol), and 0.05% Tween 20 (vol/vol), pH 8.0. The substrate, OPD was prepared at 1 mg/mL by dissolving one OPD tablet (Acros Organics) in 20 mL citrate-phosphate buffer (50 mM) to which 20 µL of 30% H_2_O_2_ was added. A 2M tetraoxosulfate-VI-acid solution was used as the stop solution. Plates were read within 5 minutes at 490 nm using a FLUorStar-Omega (BMG-Labtech) ELISA plate reader. HP concentrations were computed using the five to four parameters logistic (5/4-PL) curve-fit (MasterPlex ReaderFit v2.0, MiraiBio Group, Hitachi Solutions America, Ltd.).

### Determination of HP genotypes and protein phenotypes

We performed genotyping and phenotyping of HP in individual subjects and ascertained whether the circulatory level of HP varies with the HP1-1, HP2-1 and HP2-2 phenotypes.

For *HP* gene isoform genotyping, a forward primer, Sa5 (5′ GAGGGGAGCTTGCCTTTCCATTG 3′) and a reverse primer, Sa6 (5′ GAGATTTTTGAGCCCTGGCTGGTG 3′) were designed to amplify both *HP1* and *HP2* alleles (Supplementary Fig. 1) to detect the 1.7 kb and 3.4 kb fragments diagnostic of HP1-1 and HP2-2 genotypes, respectively (Supplementary Fig. 2a). The forward, LS1 (5′ TGAGCACTTAAGAGAGCAGGC 3′) and reverse, LS2 (5′ CTTCACATTCAGGAAGTTTATCTCC 3′) primers were designed for specific genotyping of the *HP2* allele, taking advantage of the additional sequence in the *HP2* allele not found in *HP1*. These specific primers, LS1/LS2, for the *HP2* allele were necessary because it was difficult to obtain a good amplification of the 3.4 kb fragment for the *HP2* allele in HP2-1 subjects in which the 1.7 kb fragment was also amplified. Hence, LS1/LS2 primers were designed to amplify a 1.7 kb fragment specific for the *HP2* allele and not the *HP1* allele (Supplementary Fig. 1 and Fig. 2b). Therefore, while the Sa5/Sa6 primer pair could not amplify efficiently the *HP2* allele from HP2-1 DNA for unknown reasons, it could detect the *HP1* allele, and the *HP2* allele in the HP2-1 genotype was detected by LS1/LS2 primers. The PCR mix consisted of Kapa HiFi (Kapa Biosystem) DNA polymerase (0.5 μL); 10 mM Kappa dNTP mix (0.75 μL); Kapa HiFi fidelity buffer (0.5 μL); forward and reverse primers (10 μM each); template DNA (50 ng) and distilled water to a total reaction volume of 25 μL. The PCR for LS1 and LS2 was performed at 95 °C for 3 minutes followed by 35 cycles of 98 °C for 20 seconds, 61.5 °C for 15 seconds and 72 °C for 2 minutes with a final extension step at 72 °C for 2 minutes. The PCR conditions for the Sa5 and Sa6 primers were similar to that for LS1 and LS2 but with an additional 2 mM of MgCl_2_ and at an annealing temperature of 69.2 °C or 71.5 °C. Amplified products were resolved by agarose gel electrophoresis and detected by fluorescence following staining with ethidium bromide. Subjects with only the 1.7 kb band produced with primers Sa5/Sa6 are designated as having the HP1-1 genotype, and subjects with a 1.7 kb band from both the Sa5/Sa6 and LS1/LS2 primers sets are designated as having the HP2-1 genotype while those with a 3.4 kb band from the Sa5/Sa6 and a 1.7 kb band from the LS1/LS2 primer pairs are designated as having the HP2-2 genotype (Supplementary Fig. 2a,b).

Protein HP phenotyping was carried out by western blotting and immunoprobing. Protein blotting to PVDF or nitrocellulose membrane following SDS-PAGE was performed in a Bio-Rad trans-blot cell using electro-blotting buffer (39 mM glycine, 48 mM Tris-base, 0.0375% SDS, 20% methanol and distilled water) and at a constant voltage of 30 V for approximately 15–16 hours. Polyclonal rabbit anti-human HP (Dako-Cytomation) diluted 1 in 10,000 in blocking solution (PBS containing 0.1% Tween 20 and 2–3% dried milk) was used as the primary antibody at room temperature for 2 hours or at 4 °C overnight. Alexa Fluor 488-labelled goat anti-rabbit diluted 1 in 5000 in blocking solution or HRP-conjugated goat anti-rabbit IgG diluted 1 in 10,000 in blocking solution were used as secondary antibody. The blots were then placed in blocking solution for one hour and washed in PBS-Tween. The protein bands were visualized using the Pharos FX Plus (Bio-Rad) molecular imager or chemi-luminescent detection (Supplementary Fig. 3). The western blot assay displayed the different HP phenotypes. The ~40 kDa β-chain was present in all the samples (Supplementary Fig. 3). The presence of both the α1 and α2 chains is diagnostic of the HP2-1 phenotype while the presence of only α2 or α1 indicates the HP2-2 and HP1-1 phenotypes, respectively (Supplementary Fig. 3).

### Quantitative Determination of plasma Lactate Dehydrogenase (LDH)

The level of LDH was measured by sELISA using a specific antibody (Cloud-clone Corp.) as follows. Plasma samples used for the assay were diluted 1:500 in PBS and the absorbance measured at 450 nm. LDH concentrations were calculated from the standard curve using the best-fit parameter in the MasterPlex ReaderFit v2.0 software (MiraiBio Group, Hitachi Solutions America, Ltd.).

### Quantitative Determination of plasma free-Hemoglobin (fHb)

The level of fHb was quantified by sELISA using a human fHb-specific antibody (Mybiosource) assay MBS700075 and the manufacturer’s instructions. Plasma samples were diluted 1:800 in PBS, the absorbance measured at 450 nm and fHb concentrations calculated from the standard curve using the 5-PL function in the MasterPlex ReaderFit v2.0 software (MiraiBio Group, Hitachi Solutions America, Ltd.).

### High Throughput *HP* gene sequencing

The DNA used for the *HP* gene high throughput sequencing was prepared from packed blood cells with a QIamp blood kit as described previously^51^. Primers specific to different locations on the *HP* gene were designed to produce overlapping amplicons for the entire *HP* gene (Supplementary Fig. 1) in a series of monoplex PCR reactions. The primer pairs were designed to cover extensively the promoter region of the *HP* gene; extending from about 3.4 kb upstream of the translation start site to beyond exon 7 of the *HP2* allele and exon 5 of the *HP1* allele (Supplementary Fig. 1). This covered the region 16:72083574-72095400 in the human genome database version GRCh37 or hg19. The *HP2* allele was used as the reference gene for primer design. However, the primers also recognize the corresponding region of the HP1 allele, producing shorter PCR products (Supplementary Fig. 1). The KAPA HiFi HotStart ReadyMix PCR kit (KapaBiosystems) and 50–100 ng of template DNA were used for the PCR mix.

The lyophilized primers purchased from Sigma-Aldrich were reconstituted in distilled water (dH_2_O) to a concentration of 100 μM, and then further diluted 1:10 to a working concentration of 10 μM. The final concentration of each primer in the PCR mix was about 0.3 μM in a 25 μL reaction volume.

The quality and concentration of each amplicon were analyzed by Glomax and Bioanalyzer prior to library preparation. The library was prepared and sequenced following the Illumina Nextera XT and the Illumina MiSeq machine user guides. A Phred quality score; >Q30 and paired-end sequencing was used. The FastQ output file obtained from the machine was used for further data analysis.

### Statistical Analyses

**Figure 1:**
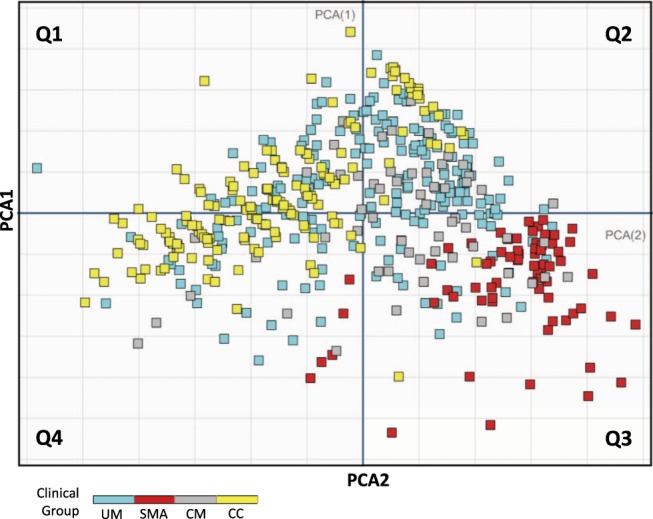
Principal Component Analysis (PCA) projection of multivariate clinical and laboratory plasma parameters of Discovery Cohort study groups at PD0. The first two principal components (PCA1 and PCA 2) were plotted in a two-dimensional scatter-plot after which the clinical group labels CC (yellow), UM (blue), CM (grey) and SMA (red) were color imposed thereafter. The projection shows the internal structure of the multivariate data while preserving its orthogonal variance. (UM) = Uncomplicated Malaria; (SMA) = Severe Malarial Anemia; (CM) = Cerebral Malaria; (CC) = healthy malaria-negative Community Controls; PD0 = at admission prior to drug treatment and/or blood transfusion.

We carried-out Principal Components Analysis (PCA) using MatLab to visualize a projection that best represents the structure of the multivariate data from all of the clinical groups of the Discovery Cohort (PD0). The multivariate data has five dimensions including the following parameters at admission: age; PCV; reticulocyte count; plasma haptoglobin and; plasma fHb). Clinical group labels and parasite-density values were excluded from the PCA analysis as it would strongly bias the results towards a malaria-positive vs. malaria-negative projection. The two orthogonal components that captured most of the variance were then plotted on two-dimensional space and clinical groups where imposed thereafter by labelling as follows: UM (blue); SMA (red); CM (grey) and; CC (yellow) (Fig. 1).

**Figure 2:**
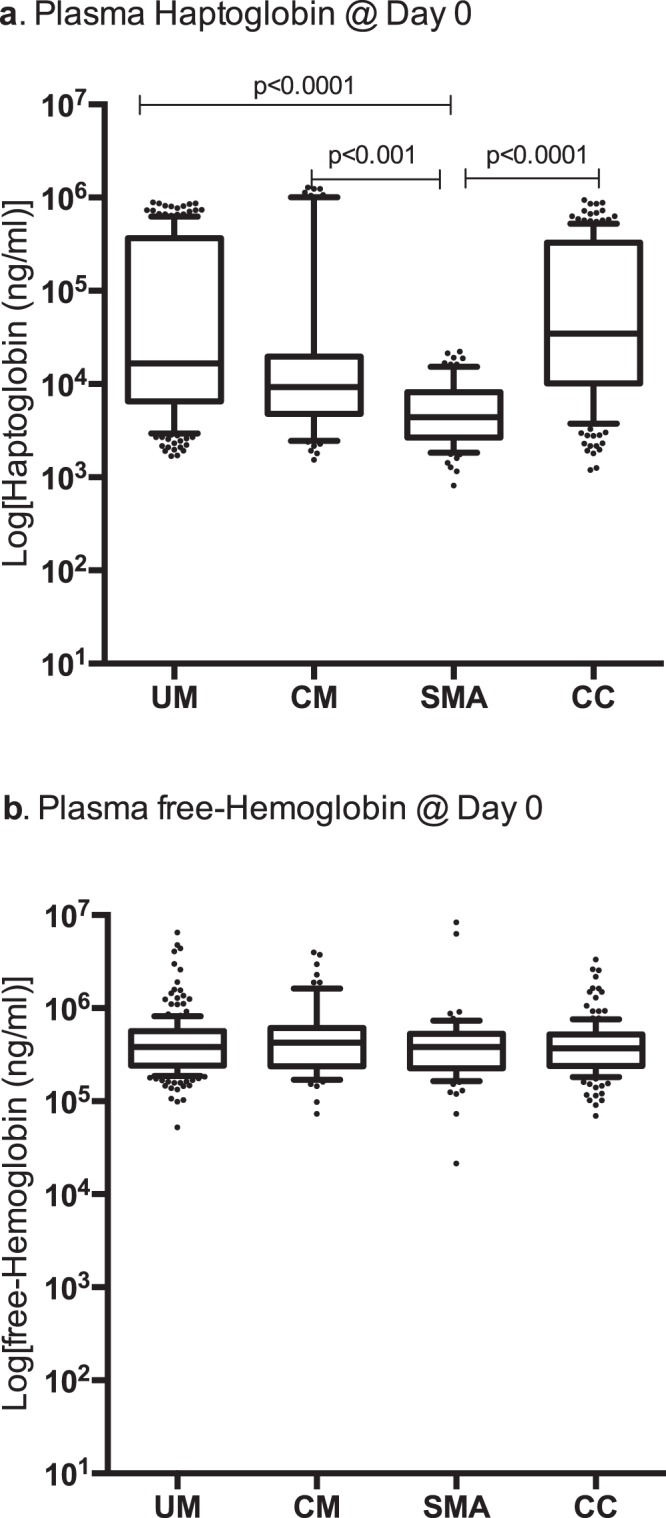
Analysis of plasma haptoglobin and plasma free-hemoglobin of discovery cohort study groups at PD0. (**a)** Box plot of Log [plasma haptoglobin (ng/ml)] at admission (PD0) in the different study groups of the discovery cohort at PD0. SMA < CC (p < 0.0001); SMA < CM (p < 0.001); SMA < UM (p < 0.0001). (**b)** Box plot of Log [plasma free-hemoglobin (ng/ml)] at admission (PD0) in the different study groups of the discovery cohort at PD0. (p > 0.05 for all comparisons). The box plots show the IQR (box); the median (box line); and the 10–90 percentile (whiskers). Non-parametric Kruskal-Wallis with Dunn’s multiple comparison correction. (UM) = Uncomplicated Malaria; (SMA) = Severe Malarial Anemia; (CM) = Cerebral Malaria; (CC) = healthy malaria-negative Community Controls; PD0 = at admission prior to drug treatment and/or blood transfusion. IQR = Interquartile Q3-Q1 Range. For discovery cohort clinical groups details see Table 1.

**Figure 3:**
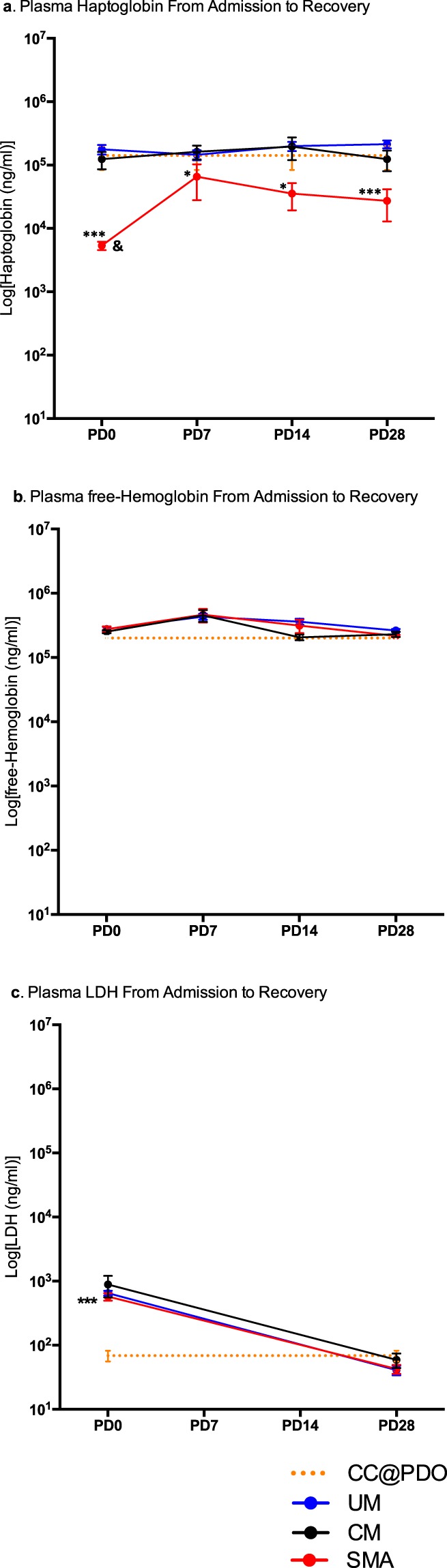
Analysis of plasma HP, fHb and LDH of validation cohort study groups from admission (PD0) through convalescence (PD7-PD14) to recovery (PD28). (**a)** Log [plasma haptoglobin (ng/ml)] from admission (PD0) through convalescence (PD7-PD14) to recovery (PD28). Asterisks show: HP SMA@PD0 < HP CC (p < 0.001); HP SMA@PD7 < HP CC (p < 0.05); HP SMA@PD14 < Plasma HP CC (p < 0.05); HP SMA@PD28 < HP CC (p < 0.001). Ampersand (&) shows: HP SMA@PD0 < HP SMA@PD28 (p < 0.01) (**b)** Log [plasma free-hemoglobin (ng/ml)] from admission (PD0) through convalescence (PD7-PD14) to recovery (PD28). **c)** Log [plasma LDH (ng/ml)] from admission (PD0) through convalescence (PD7-PD14) to recovery (PD28). Plasma LDH in all malaria-positive groups at PD0 > CC (p < 0.001). Plots show mean (circles) and standard error of the mean (whiskers). Asterisk: *(p < 0.05); **(p < 0.01); ***(p < 0.001). Non-parametric Kruskal-Wallis with Dunn’s multiple comparison correction (between group comparisons). Paired non-parametric Wilcoxon tests were used to compare paired data within a disease group. For validation cohort clinical group details see Table 1. (UM) = Uncomplicated Malaria (blue line); (SMA) = Severe Malarial Anemia (red line); (CM) = Cerebral Malaria (black line); (CC) = healthy malaria-negative Community Controls (yellow dotted-line); HP = haptoglobin; fHB = free hemoglobin; LDH = lactate dehydrogenase; PD0 = at admission prior to drug treatment and/or blood transfusion; PD7 = Plasma sample collected at the 7^th^ day after PD0; PD14 = Plasma sample collected at 14^th^ day after PD0; PD28 = Plasma level collected on 28^th^ day after PD0 (recovery).

**Figure 4:**
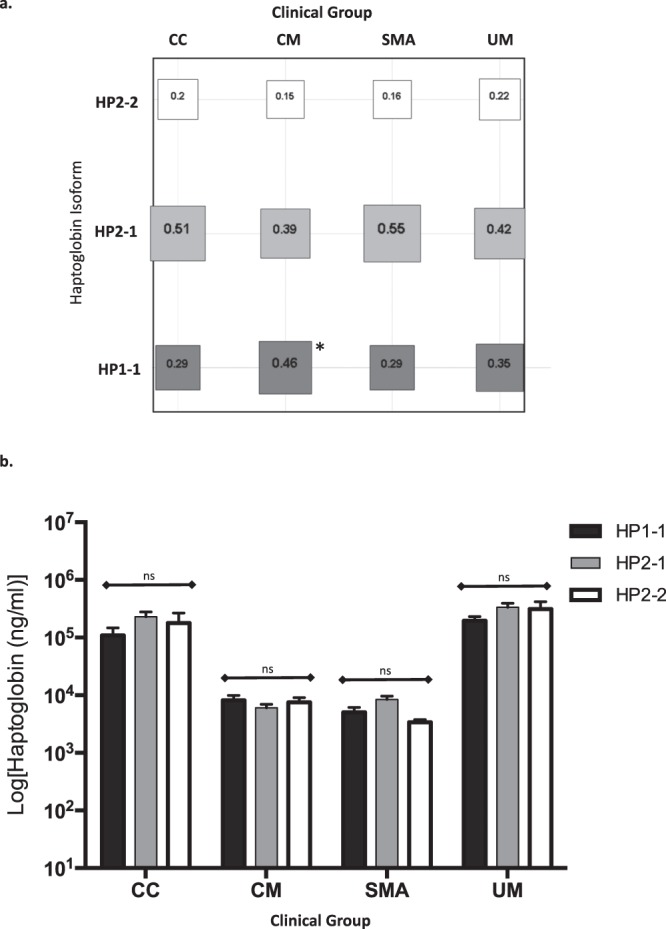
Haptoglobin isoform frequencies and plasma HP of HTS Cohort study groups. (**a**) Frequency of haptoglobin isoforms HP1-1 (dark-grey square); HP2-1 (grey square) and HP2-2 (white square) for the clinical groups. Sizes of the square are drawn to scale and depicts the frequency of the isoform written with the square. HP1-1, HP2-1 and HP2-2 are HP phenotypes. *The HP1-1 isoform is associated with CM (p = 0.038) with an estimated OR of 2.1 (1.04–4.4). (**b)** Bar plot of Log [plasma HP (ng/ml)] per isoforms HP1-1 (black bar); HP2-1 (grey bar) and HP2-2 (white bar) for the clinical groups. Bar plot shows mean and standard error of the mean (whiskers). Non-parametric Kruskal-Wallis with Dunn’s multiple comparison correction; ns = not significant (p > 0.05). (UM) = Uncomplicated Malaria; (SMA) = Severe Malarial Anemia; (CM) = Cerebral Malaria; (CC) = healthy malaria-negative Community Controls. For HTS Cohort clinical group details see Table 1.

Unpaired non-parametric Mann-Whitney tests were used to compare clinical demographic data between two disease groups (Table 1). Non-parametric Kruskal-Wallis tests were performed to determine differences between disease groups using Dunn’s multiple comparison correction (Figs 2–4). Paired non-parametric Wilcoxon tests were used to compare paired data within a disease group (Fig. 3). Non-parametric Spearman tests were used to correlate the levels of fHb and HP with parasite density in malaria-positive clinical groups (Supplementary Table 1). Linear regression was used to determine independency of HP levels with age in the malaria-negative CC group (Supplementary Fig. 4).

**Table 1:** Clinical Characteristics of Cohorts Study Groups.

Discovery Cohort	Clinical Groups [Total N = 545]			
	UM	CM	SMA	CC
**N (%)**	216 (40)	69 (12)	86 (16)	174 (32)
**Age (months)** Median (IQR)	42 (24–78)	45 (29–60)	29 (20–44)	84 (60–120)
**Sex: F/M**	88/128	40/29	39/47	95/79
**PCV (%)** Median (IQR)	29 (24–34)	27 (21–31)	13 (11–15)	35 (33–37)
**Parasite Density** Median (IQR)	29940 (2464–52021)	10428 (1280–58844)	21883 (1222–47847)	N/A
**Validation Cohort**	**Clinical Groups [Total N = 166]**			
	**UM**	**CM**	**SMA**	**CC**
**N (%)**	71 (40)	40 (21)	36 (19)	19 (10)
**Age (months)** Median (IQR)	36 (21–73)	47 (36–62)	39 (27–61)	48 (32–72)
**Sex: F/M**	27/44	17/23	8/28	8/11
**PCV (%) PD0** Median (IQR)	32 (27–35)	25 (23–30)	13 (11–15)	34 (32–35)
**PCV (%) PD28** Median (IQR)	35 (29–40)	34 (27–40)	34 (27–37)	35 (27–40)
**Parasite Density** Median (IQR)	27429 (1901–54680)	5590 (1314–81923)	32131 (2487–103822)	N/A
**HTS Cohort**	**Clinical Groups [Total N = 401]**			
	**UM**	**CM**	**SMA**	**CC**
**N (%)**	153 (38)	66 (16)	81 (20)	101(26)
**Age (months)** Median (IQR)	38 (21–71)	43 (28–60)	29 (22–45)	80(48–96)
**Sex: F/M**	73/80	31/35	39/42	58/43
**PCV (%)** Median (IQR)	31 (24–35)	26 (21–31)	14 (11–15)	35 (33–36)
**Parasite Density** Median (IQR)	27717 (2431–53333)	20099 (1280–55688)	16089 (1093–47221)	N/A

Discovery Cohort study groups: no significant differences in PCV, age and parasite density (p > 0.05). Validation Cohort study groups: used for the follow-up through convalescence to recovery time point analysis; no significant differences in PCV, age and parasite density (p > 0.05). High Throughput Sequencing (HTS) Cohort Study Groups: A total of 401 subjects *HP* gene were sequenced; no significant differences in PCV, age and parasite density (p > 0.05).

Non-parametric unpaired Mann-Whitney test. (UM) = Uncomplicated Malaria; (SMA) = Severe Malarial Anemia; (CM) = Cerebral Malaria; (CC) = healthy malaria-negative Community Controls. The age, PCV and parasite density were presented as Median and Interquartile Range. IQR = Interquartile Range; % = Percentage; N = Number; N/A = Not Applicable; PCV = Packed Cell Volume. (F/M) = Male/Female; (N/A) = Not Applicable; (HTS) = High Throughput Sequencing.PD0 = Plasma day zero at admission; PD28 = Plasma day 28 (recovery).

**Figure 5:**
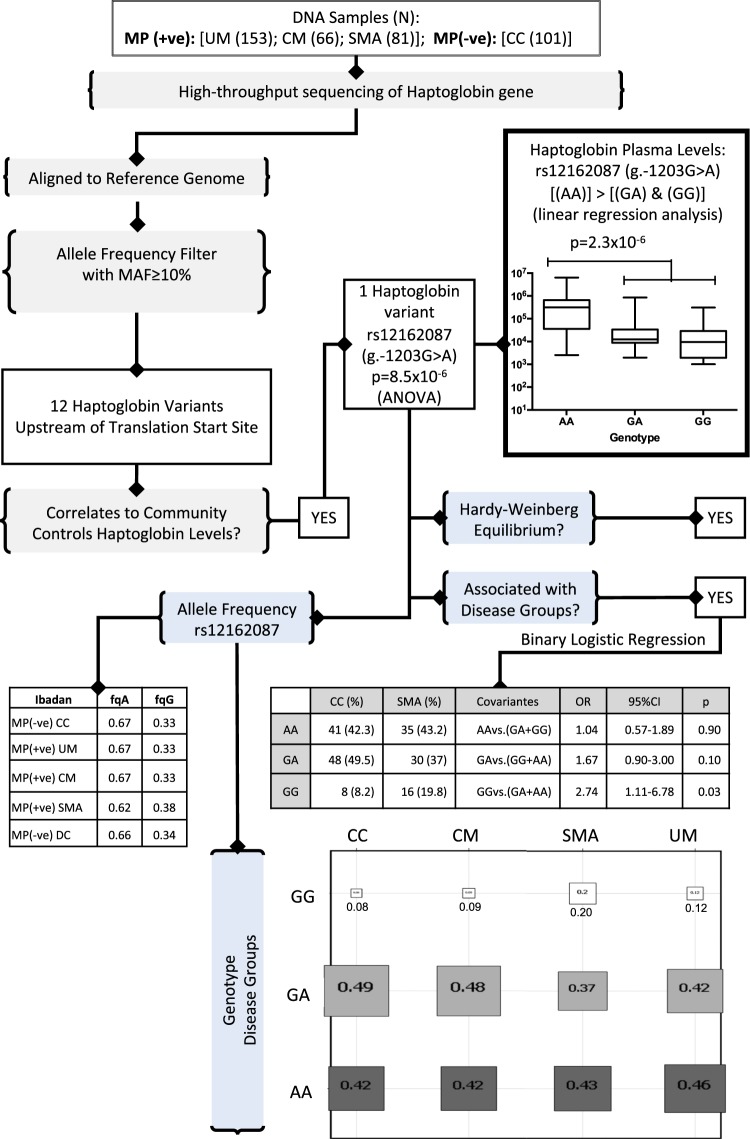
Flow chart showing the variant filtering strategies and association studies used in the Ibadan HTS-Cohort. High throughput sequencing of Haptoglobin gene has been performed on 401 samples. We used an allele frequency filtering to select the variants with a MAF ≥ 10%. ANOVA and linear regression analysis were then used to evaluate their association with the haptoglobin level in CC group. One variant rs12162087 was selected upon this association. GenePop software was used to determine the Hardy Weinberg equilibrium in control group. We performed a binary logistic regression to test the association of this variant and SMA. The allele and the genotype frequencies for rs12162087 are indicated for each disease group. (UM) = Uncomplicated Malaria; (SMA) = Severe Malarial Anemia; (CM) = Cerebral Malaria; (CC) = healthy malaria-negative Community Controls; (N) = number of subjects; MP(+ve) = malaria parasite positive; MP(−ve) = malaria parasite negative, MAF = Minor allele frequency. For HTS Cohort clinical group details see Table 1 and details for variants see Table 2.

For the genomic data and the identification of variants, FASTQ output files were aligned to the reference genome using Burrows-Wheeler Aligner (BWA). Samtools was used on the BAM file to produce the output VCF file, containing the variants. This was used for downstream computation such as the measure of association (Odds Ratio; OR), 95% CI and genotype frequency. Minimum Allele Frequency greater or equals 10% (MAF ≥ 10%) were used to select variants (Fig. 5). We used Mendelian randomization with the assumption that the inheritance of SNP is essentially random, to determine the effect of the SNP on HP level and disease. The CC subjects were genotyped for rs12162087 polymorphism and the frequency of each genotype (AA, GA, GG) was determined together with the HP levels (Fig. 5). Each participant genotype for the observed SNP were measured and the frequency determined relative to the CC. chi-Square tests were used to determine whether the genotype distributions in the control group were conformed to the Hardy-Weinberg equilibrium (Fig. 5) GenePop software (web version 4.2 option 1). The genotypes effect on HP levels were estimated by ANOVA and linear regression analysis while univariate analyses of HP variants (binary logistic regression) were carried out with SPSS version 10.1 to examine association between the genotype and SMA (Fig. 5).

## Results

### Study Participants

The discovery cohort (Table 1) consists of 545 subjects in total with 371 malaria-positive patients with either UM, CM or SMA whose plasma samples were collected at acute onset of the disease (PD0) and 174 malaria-negative healthy community controls (CC).

The validation cohort (Table 1) consists of 147 malaria-positive patients with either UM, CM or SMA and 19 healthy malaria-negative community controls. This cohort was independently recruited to validate the data obtained at PD0 in the discovery cohort and more importantly to observe trends within the measured parameters through convalescence to recovery.

The HTS cohort (Table 1) consists of a total 401 subjects with 300 malaria-positive patients and 101 healthy malaria-negative community controls drawn from both the discovery and validation cohorts and whose HP genes were sequenced to determine whether an association between genetic variation, clinical syndromes and observed plasma HP levels exist.

As expected, PCV values were lowest in the SMA clinical group. In SMA cases, low PCV at recruitment (PD0) was attributed to the anemia as the consequence of the malaria infection. In all clinical malaria-positive groups receiving anti-malaria treatment the PCV returned to normal at recovery (PD28) Table 1. The median age of children with SMA were slightly lower but not significantly different (p > 0.05) to other disease groups and the healthy control (CC). It is well-known within the sub-Saharan West African holoendemic high-transmission malaria region that SMA is observed in younger children^5,39,40,48,49^. Although G6PD deficiency has been associated with protection against SMA (G- males only) in a previous cohort from Ibadan^50^, we did not observe this in our present cohort (data not shown).

The mean parasite densities were not significantly different among the malaria-positive groups; the CM, SMA and UM. Females and males were reasonably represented in this study. The clinical demographics of the subjects within the Discovery Cohort (Table 1), Validation Cohort (Table 1) and HTS Cohort (Table 1) were not different (p > 0.05).

### Children presenting with SMA have significantly lower plasma HP than any other study group at hospital admission in the discovery cohort

To explore the structure of the multivariate data and to obtain the most visually useful projection of its variance in two-dimensional space, we created a scatter-plot with the two most informative principal components coefficients of all study groups in the discovery cohort (Fig. 1) followed by imposing their respective group label UM (blue), SMA (red), CM (grey) and CC (yellow). To facilitate visualization of the structure of the data the two-dimensional PCA space is divided clock-wise in four quadrants (Fig. 1: Q1, Q2, Q3 and Q4). Children with SMA mostly aggregated inside the Q3 quadrant and in a manner that is more homogeneous than that of the other groups (Fig. 1 – Q3 red). There is little overlap of children with SMA (red) in Q3 and those within the UM (blue), CM (grey) and CC (yellow) groups (Fig. 1). Children with UM (blue) spread more heterogeneously across all quadrants with the majority in Q2, Q1, and Q4 respectively (Fig. 1). Children with CM (grey) aggregated predominantly within Q2 and Q3 and overlap greatly with the UM (blue) group in this projection of the data. Children in the CC (yellow) group spread across Q1, Q2, Q4 (Fig. 1) and clearly overlap with the UM (blue) group in this projection of the data (Fig. 1). Overall, the PCA projection shows the SMA group as more homogeneous and clearly amenable of linear separation from the rest of the groups (Fig. 1). Observed overlap of variance of UM, CM and CC groups in the PCA projection suggests that non-linear methods are required.

The plasma HP levels at admission in the SMA group are significantly lower when compared to the levels in the other malaria groups SMA < CC (p < 0.0001); SMA < CM (p < 0.001); SMA < UM (p < 0.0001) (Fig. 2a). All children with SMA presented with hypo-haptoglobinemia with plasma HP levels below 2 × 10^4^ ng/ml, median of 4 × 10^3^ ng/ml and mean of 6 × 10^3^ ng/ml (Fig. 2a).

The low plasma HP level in the SMA group does not appear to be dependent on the fHb level since the levels of fHb did not differ among the malaria groups (Fig. 2b). HP plasma levels do not correlate with age in the healthy malaria-negative CC group (Supplementary Fig. 4).

### Plasma HP level in children with SMA remained low through convalescence to recovery in the validation cohort

The validation cohort was used to test whether the low HP levels at PD0 observed in the discovery cohort would remain low through convalescence to recovery. As shown in the discovery cohort (Fig. 2a), HP levels were significantly lower at PD0 in SMA (mean of 7 × 10^3^ ng/ml) compared to the other clinical groups CM, UM and CC (p < 0.001) (Fig. 3a). Overall, the HP levels in the SMA group remain significantly lower at all time points compared to levels in both other malaria groups and the parasite-negative CC group (Fig. 3a). Although SMA HP levels at PD28 are significantly higher than that at PD0 (p < 0.01), these levels at recovery still remain significantly lower (mean of 5 × 10^4^ ng/ml) than those of the UM, CM and CC group (mean 10^5^ ng/ml) (p < 0.001) (Fig. 3a). The validation cohort also shows that SMA HP plasma level remains low through convalescence to recovery (Fig. 3a) despite stabilized levels of plasma fHb (Fig. 3b) and decreasing levels, to normal range, of LDH at recovery (Fig. 3c). Unlike the SMA group, plasma HP levels through convalescence to recovery in both the CM and the UM groups were not different to those of the CC group (Fig. 3a).

### Plasma fHb and LDH levels in children presenting with SMA were not different to those of other malaria groups in the validation cohort

The levels of both fHb and LDH through convalescence to recovery in the SMA group in the validation cohort do not appear to be higher than those of the UM and CM groups (Fig. 3b,c). LDH levels returned to normal at recovery in all malaria groups; levels at PD28 were not different to the level in the CC group (Fig. 3c).

### Plasma HP and fHb levels correlate differently with malaria parasite density

The plasma HP levels significantly inversely correlated with parasite density in the UM group (Supplementary Table 1). On the other hand, there was no correlation between fHb and parasite density in the UM group (Supplementary Table 1). No correlations exist between plasma HP and parasite density in the CM and SMA groups (Supplementary Table 1). A significant direct correlation (p = 0.03) between fHb and parasite density was detected in the SMA group, not reciprocated in the CM group (Supplementary Table 1).

### HP isoform phenotype does not show association with SMA or plasma HP level in the HTS cohort

We found no association between HP isoform phenotype and SMA (Fig. 4a). The low HP levels observed in the SMA are equally independent of HP isoform (Fig. 4a,b). The plasma levels of the different isoform phenotypes within a disease group are not different (Fig. 4b).

The HP1-1 isoform is associated with CM (p = 0.038) with an estimated OR of 2.1 (1.04–4.4); there is a higher frequency of HP1-1 in the CM group (0.46) compared to the CC (0.29), UM (0.35) and SMA (0.29) groups (Fig. 4a). The observed association between CM and HP1-1 does not appear to be related to plasma HP level within the isoforms (Fig. 4b).

### A Single Nucleotide Polymorphism (rs12162087) (g.-1203G > A) is associated with low HP levels and SMA disease in the HTS cohort

Twelve variants with MAF ≥ 10% found in the *HP* gene, all located 3.4 kb upstream of the translation start site, were analyzed (Table 2) as summarized in Fig. 5. The HP plasma level of healthy malaria-negative CC group was used to establish the association with the rs12162087 variant as HP plasma level at PD0 in the malaria-positive disease clinical groups might not actually reflect their actual congenital level (Fig. 5). We used ANOVA and linear regression analysis to detect a SNP that correlates with HP circulatory level in the healthy subjects, the CC group, and found rs12162087 (g.-1203G > A; 16:72087307) to be significantly associated (p = 8.5 × 10^−6^) (Fig. 5 and Table 2). The GG genotype is associated with very low HP plasma level; the AA genotype has the highest level of plasma HP while the plasma HP level in the GA genotype is intermediate (Fig. 5).

**Table 2:** *HP* gene variants observed in the HTS cohort with MAF ≥ 10%.

^#^CHRO	POS	AF	REF	ALT	ID	HP-CC
16	72085561	0.83	T	A	rs9924964	0.2365
16	72086460–72086462	0.19	T	TTC	Unknown	0.6478
16	72086469–72086476	0.18	ACACACAC	TCACACAC, ACACAC, ACACACAT	Unknown	0.7426
16	72086534	0.83	A	T	rs7201866	0.1654
16	72086555	0.66	T	C	rs7203426	0.0948
16	72086741	0.63	GCCTGA	GCCTGG, ACCTGG	Unknown	0.0543
16	72087058	0.17	T	C	rs28639994	0.1162
**16**	**72087307**	**0.66**	**G**	**A**	**rs12162087**	*8.5 **×** **10**^**−6**^
16	72088227	0.12	T	C	rs5466	0.6105
16	72088418	0.44	TGAC	AGAC, TGAG, TTAC	Unknown	0.1587
16	72088461	0.14	A	C	rs5471	0.2365
16	72088467	0.47	A	G	rs5472	0.0557

Genetic variations on the sequenced *HP* gene with MAF ≥ 10% and their association with HP plasma levels in the healthy malaria-negative Community Controls (HP-CC).

^#^CHROM = Chromosome Number; POS = Position; AF = Allele Frequency; REF = Reference Allele; ALT = Alternative Allele; ID = Variant ID; Unknown = Previously Unknown Variant; HP = Haptoglobin; CC = malaria-negative Healthy Community Controls; *rs12162087 is significantly associated with HP levels in Healthy Community Controls (p < 8.6 × 10^−6^ using ANOVA with Bonferroni correction**)**.

To evaluate whether rs12162087 is associated with malaria syndromes, we performed a genetic association study. The analysis revealed a significant association between SMA and rs12162087 (p = 0.03). The (Homo-Ref: GG) genotype frequency is higher in SMA (19.8%) compared to CC (8.2%) with an OR of 2.74 (95% CI = 1.11–6.78). All together our findings in the Nigerian cohort show that the genotype HP rs12162087 GG is more common in SMA and is associated with lower HP levels (Fig. 5).

## Discussion

Since the discovery of haptoglobin in 1938^51^, its levels have been used as a marker of hemolysis^20^. The causes and consequences of low plasma HP in clinical malaria are difficult to dissect. Genetic factors, inflammatory acute phase responses and hemolysis-induced anemia have been suggested to play a role in the low level of HP observed in life-threatening childhood SMA^28,44,45^, a severe malaria syndrome that requires urgent blood transfusion. A common assumption has been that low HP level in SMA results from parasite-induced hemolysis^31,46^. However, by measuring the levels of HP, fHb and LDH on hospital admission, during convalescence to recovery, our study shows that low HP levels are associated with susceptibility to SMA.

Our study contributes to the understanding of why some children go onto developing life-threatening SMA while other children the symptoms are restricted to UM. In the present study, HP levels at acute onset were lowest in children with SMA (one order of magnitude lower) and also lower than normal in the CM group, an observation that appears consistent with the hemolysis-induced hypo-haptoglobinemia hypothesis^18,20–22^. However, while the plasma HP levels recovered in children with UM and CM, most children presenting with SMA had a low HP level that remained low during convalescence to recovery, suggesting that hypo-haptoglobinemia was already present in these patients prior to the malaria episode and the acute onset of SMA.

It is intractable to carry-out a large clinical study where HP levels are measured at the initial day of infection, especially in holoendemic high-transmission malaria settings such as ours. To tackle this challenge, we focused on the disease trajectory of the malaria-positive clinical groups from day of diagnosis (PD0) through convalescence (PD7, PD14) to recovery (PD28) in order to establish a possible genetic etiology of the observed hypo-haptoglobinemia. In our study, children recovery at four weeks (PD28) post-diagnosis is strictly defined by clinical and laboratory parameters such as return to normal values of PCV, fHb, LDH and reticulocyte counts. Our definition of recovery at PD28 is also consistent with previous studies that show that hemoglobin levels as low as 5.0 g/dL in SMA returns to normal 2–3 weeks after commencement of treatment^52^. Similarly, other studies have reported that as parasite disappears from the blood, either by antimalarial therapy or host mechanism, both hemoglobin level and bone marrow morphology returns to normal^53^ 2–3 weeks which is within the convalescence phase^54^. Furthermore, in acquired hypo-haptoglobinemia, induced by malaria or hemolytic disease, HP levels return to normal 2 weeks after the commencement of antimalarial treatment^32,47^. In line with these previous findings and coupled with the clinical and laboratory evidence of recovery provided in our study, the PD28 recovery time-point is sufficient for the HP level and other clinical parameters to normalize as it happens in the UM and CM clinical groups. By measuring HP levels at these time-points we have been able to study the role of congenital hypo-haptoglobinemia over that of other etiologic causes. Although hypo-haptoglobinemia can be induced by hemolysis, a genetic contribution to this phenomenon can also be equally important as our data suggest. Our study provides evidence that the etiology of hypo-haptoglobinemia observed in children with acute-onset SMA could be due to genetic factors as HP levels fail to rise after the underlying cause of pathology has been successfully treated and the children recovered four weeks after diagnosis.

The evidence that the rs12162087 (g.-1203G > A) genetic variant, with an observed frequency of 0.67 in this predominantly Yoruba Ibadan’s cohort, is associated with plasma HP levels in the malaria-negative CC group is fairly compelling. First, the association is determined within a single cohort, the malaria-negative well-children cohort, reducing the possibility that confounders play a role. Second, this variant is the only common variant that has such strong evidence suggesting little confounding acting across SNPs. Third, the variant is not out of Hardy-Weinberg equilibrium, suggesting that it is well genotyped by our HTS protocol and there is not stratification bias in the CC group. We show that children with rs12162087 GG genotype showed a risk of 2.74 to develop SMA compared to those with the rs12162087 GA and AA genotypes.

This is the first time a variant in the distal upstream region of the *HP* gene is reported to be associated with both circulatory HP level and the disease, SMA^29,55^. The rs12162087 (g.-1203G > A) variant has not been associated with another trait before. The variant is upstream of the transcriptional start site of the *HP* gene, raising the likelihood that it might be a regulatory variant affecting gene expression. Furthermore, in HaploReg this variant is highlighted as enhancer histone marks and it is associated with eQTL. We suggest that there is a possible interaction between the distal upstream region and the proximal upstream (promoter) region of the HP gene through DNA looping that could affect the expression of the protein.

Aside the hypo-haptoglobinemia observed in SMA patients, we also showed that plasma HP level is independent of HP isoform phenotype. Replicating the findings of a previous study in Ghana^56^, we found that the Hp1-1 isoform is significantly more prevalent in children presenting with CM than in the CC group in our Nigerian cohort. However, our larger sample size than the previous study^56^ did not detect any significant differences between Hp1-1 isoform in the SMA when compared to CC. Consistent to a study carried out in Kenya^57^, we found that the Hp2-1 isoform does not confer protection against SMA. In the Kenyan cohort^57^, the Hp2-2 isoform is associated with the greatest risk of CM and Hp2-1 with greatest protection against CM. In our west-Africa CM cohorts we found that Hp1-1 was more common in the CM patients, implying a potential for greater risk factor, whilst Hp2-1 and Hp2-2 seem to be associated with protection. Further multi-site studies are needed to dissect the differences between these west- and east- African cohorts and to investigate if there is a correlation between HP isoform and the reported rs12162087 variant.

The reported long heterozygous and homozygous deletions of the *HP* gene known to be associated with hypo-haptoglobinemia^28,36^ are rare in this population. This deletion was found in only 1 out of 401 subjects whose *HP* genes were sequenced. These deletions appear not to be the major cause of hypo-haptoglobinemia in this predominantly Yoruba population.

Our data shows that the degree of intravascular hemolysis in the SMA group is not different to that of other malaria positive groups. In acute-onset life-threatening childhood SMA rapid PCV decrease^58^ is predominantly driven by increased clearance of uninfected erythrocytes by the reticulo-endothelial system^49^ as intravascular hemolysis of infected erythrocytes due to schizogony cannot explain by itself this dramatic change. In line with our previous SMA study^49^, we suggest that, in the cohorts with baseline low plasma HP and Hb, particularly the SMA group, clearance of non-infected erythrocytes is driven by the reticulo-endothelial system in the spleen through an as yet unknown CD8(+) T-cell-dependent mechanism^49^. This splenic clearance of erythrocytes could be responsible for the low Hb level in the SMA group. This further indicates that extravascular hemolysis is implicated more strongly in the pathology of SMA rather than intravascular hemolysis.

It is unknown why some children develop severe forms of malaria such as SMA whilst in others the infection is restricted to the mild form of the disease. Our study suggests that a low circulatory HP level is a risk factor for the occurrence of SMA in contrast to the other malaria syndromes studied. Children bearing the genotype at risk rs12162087 g.-1203G/G had one order of magnitude lower HP levels which is consistent also with plasma HP levels observed to be low in SMA children at disease onset and at recovery.

Although multi-site studies within Africa and outside Africa, with populations under different malaria burden, are needed to further dissect the relationship between of *HP* genotypes with hypo-haptoglobinemia, our findings provide evidence that the hypo-haptoglobinemia phenotype and the presence of the rs12162087 g.-1203G/G genotype could be used as predictors for acute onset SMA. We expect that our findings will prompt these multi-site studies, not only to replicate association, but to dissect what other characteristics of other clinical settings can reinforce the significance of genetic control of HP plasma levels in determining acute onset severe anemia phenotypes including that induced by malaria.

Finally, our study contributes to the development of risk assessment algorithms to identify children with higher risk to develop SMA, which is lethal without urgent access to safe blood transfusion, this being the case in all (urban, peri-urban and rural) areas in high-transmission malaria holoendemic regions in sub-Saharan Africa. Improved risk assessment algorithms will also have a substantial impact on the use and management of health-care resources such as blood banks across low- and middle-income regions of the World under malaria burden.

## Electronic supplementary material

Supplementary Information
