# Supplementary material for: Low plasma haptoglobin is a risk factor for life-threatening childhood severe malarial anemia and not an exclusive consequence of hemolysis
Source: Sci Rep. 2018 Dec 3;8:17527. doi: 10.1038/s41598-018-35944-w (PMC6277387; doi:10.1038/s41598-018-35944-w)
Supplement: Supplementary file 1 — Supplementary Information [file 41598_2018_35944_MOESM1_ESM.pdf]

**Low plasma haptoglobin is a risk factor for life-threatening childhood severe malarial anemia and not an exclusive consequence of hemolysis.**

Samuel Eneọjọ Abah [1#], Florence Burté [1#], Sandrine Marquet [2,3#], Biobele J. Brown [4,5#], Francis Akinkunmi [4], Gbeminiyi Oyinloye [4,5], Nathaniel K. Afolabi [4,5], Samuel Omokhodion [4,5], Ikeoluwa Lagunju [4,5], Wuraola A. Shokunbi [5,6], Mats Wahlgren [7], Hélia Dessein [2], Laurent Argiro [2], Alain J. Dessein [2], Boris Noyvert [1], Lilian Hunt [1], Greg Elgar [1], Olugbemiro Sodeinde [4,5,8], Anthony A. Holder [1], Delmiro Fernandez-Reyes* [4,5,8]

**1** Francis Crick Institute, 1 Midland Road, London, NW1 1AT, United Kingdom.

**2** Aix-Marseille University, Inserm GIMP, Labex ParaFrap, Marseille, 13385, France.

**3** Aix-Marseille University, Inserm Laboratoire TAGC/U1090, Marseille, 13288, France.

**4** Department of Paediatrics, College of Medicine, University of Ibadan, University College Hospital, Ibadan, Nigeria.

**5** Childhood Malaria Research Group, College of Medicine, University of Ibadan, University College Hospital, Ibadan, Nigeria.

**6** Department of Haematology, College of Medicine, University of Ibadan, University College Hospital, Ibadan, Nigeria.

**7** Department of Microbiology, Tumour and Cell Biology, Karolinska Institutet, Stockholm, Sweden.

**8** Department of Computer Science, Faculty of Engineering, University College London, Gower Street, London, WC1E 6BT, United Kingdom.

**#** Contributed equally to this work. *****Corresponding Senior Author.

**Supplementary Table 1.**

Correlations analysis on day of recruitment (PD0) between:

**a)** Plasma Haptoglobin (HP)

**b)** Plasma free-Hemoglobin (fHb) with Parasite Density (PD)

| Clinical Group | a) HP & Parasite Density at PD0 | | | b) fHb & Parasite Density at PD0 | | |
|---|---|---|---|---|---|---|
| | r | 95% CI | p value | r | 95% c.i. | p value |
| UM | -0.2423 | -0.4682 to 0.01339 | 0.0275* | -0.1025 | -0.4611 to 0.2849 | 0.2984 |
| CM | -0.2125 | -0.5200 to 0.1457 | 0.1138 | 0.1974 | -0.3269 to 0.6288 | 0.2224 |
| SMA | -0.1906 | -0.5046 to 0.1680 | 0.2804 | 0.3829 | -0.08593 to 0.7128 | 0.0300* |

Non-parametric Spearman test. *=Statistically significant, P<0.05
UM=Uncomplicated Malaria; CM=Cerebral Malaria;
SMA=Severe Malarial Anemia

**Supplementary Figure 1.**

Gene structure of the *HP2* and *HP1* isoform haptoglobin alleles and notation of primer mapping used for genotyping and primer location for PCR amplicon sequencing.

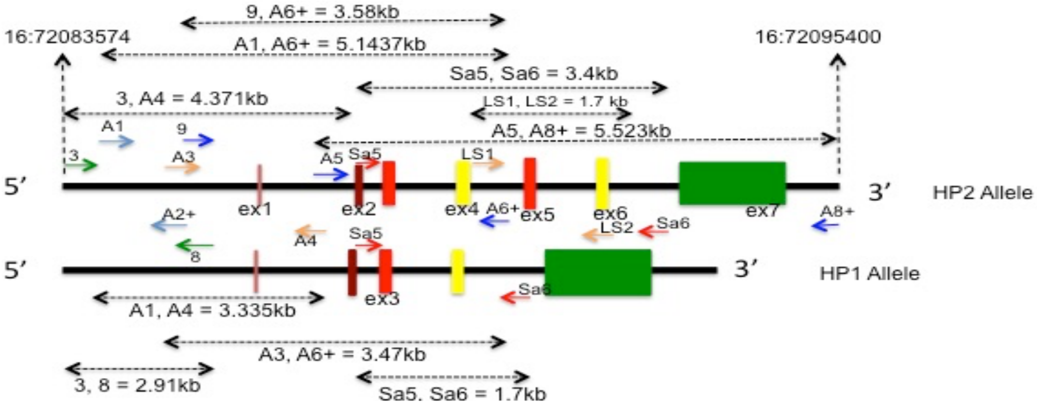


The structure of the HP1 and HP2 haptoglobin alleles. Short single arrowheads indicate the primers positions for amplification; the long dotted double arrowheads indicate the DNA fragment size amplified by the primer pairs. The positions for the pairs of primers Sa5 and Sa6 are indicated by the red arrows. The LS1and LS2 primers, which are specific for the *HP2* allele are indicated by the yellow arrows. Exons (ex) are labeled 1 to 7. Both exons 3 and 4 are duplicated in the *HP2* allele and the *HP2* allele is 1.7kb longer than the *HP1* allele due to this duplication. The combinations of these primers were used for specific genotyping of *HP* among subjects.

**Supplementary Figure 2**
Haptoglobin isoforms genotyping

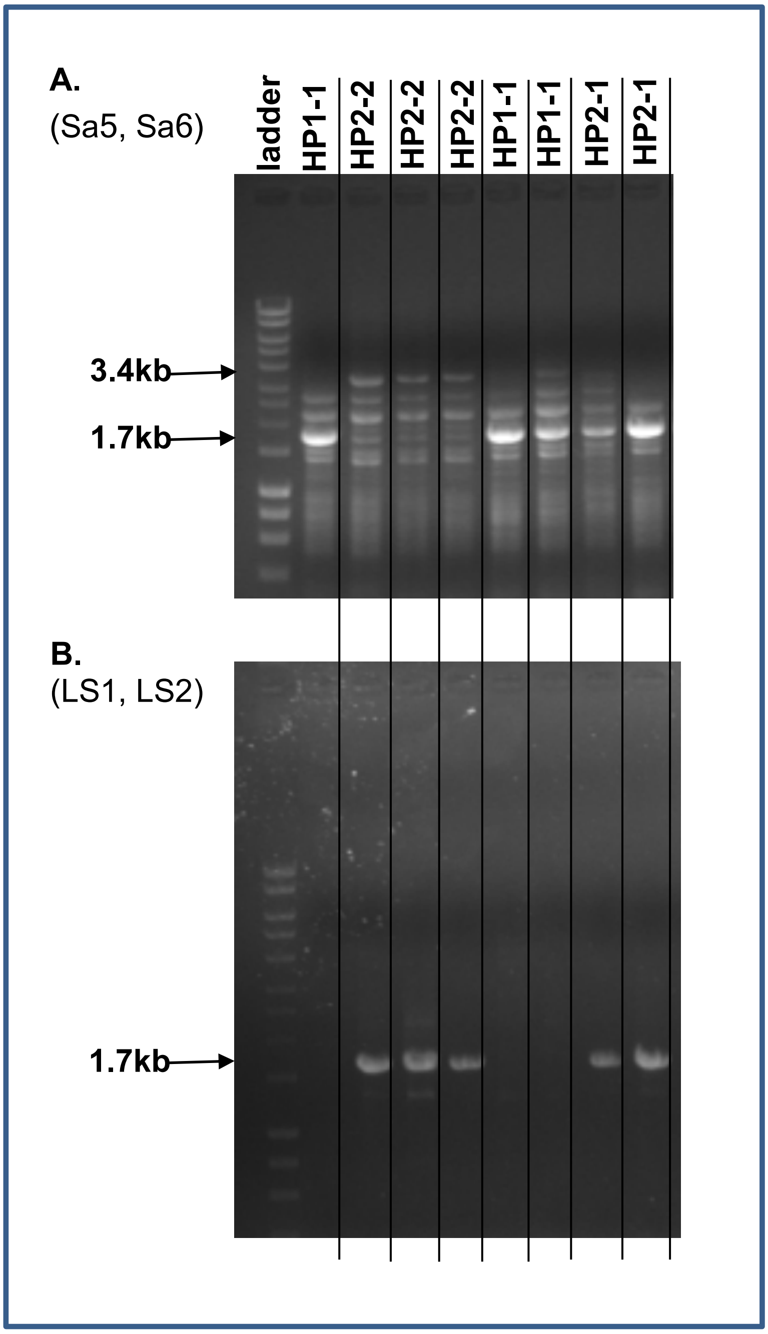


**a.** The primers Sa5/Sa6 were designed to produce a 3.4kb band for *HP2* isoform allele and a 1.7kb band for the *HP1* isoform allele. However, the 3.4kb band was not well identified, particularly in the *HP2-1* genotype. **b.** Therefore to detect the *HP2* isoform allele in both HP2-1 and HP2-2 genotypes we amplified a 1.7kb band using the LS1 and LS2 primers which are HP2-specific. The combined results from A and B were then used to establish the genotype (see top of A. gel). See Supplementary Figure 1 for primer location. Each lane shows PCR amplification of eight different subjects with gels A. and B. mirroring the same subject. (image shows full raw gels)

**Supplementary Figure 3.**
Haptoglobin isoforms phenotyping

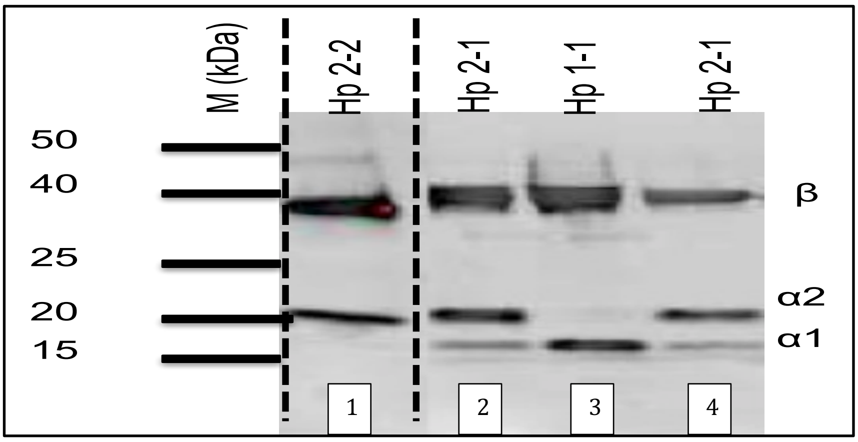


Western blot analysis of HP isoform phenotypes. The β, α2 and α1 represent the beta, alpha 2 and alpha 1 HP chains respectively. The HP2-2 phenotype has only the α2 chain while the HP1-1 phenotype has only the α1 chain; the HP2-1 phenotype has both α2 and α1 chains. M (kDa) indicates the migration of molecular weight markers. The figure shows the results for four different subjects. Briefly, SDS-PAGE of plasma from different subjects (using Bio-Rad Precision-Plus Protein Marker as ladder) was blotted and immune-probed with a polyclonal rabbit anti-human HP antibody. To visualize protein bands, we used a Bio-Rad Pharos FX$^{TM}$ Plus Molecular Imager and Bio-Rad PD-Quest$^{TM}$ 2-D Analysis Software (red-spot shows scanning alignment). The Bio-Rad PD-Quest$^{TM}$ 2-D Analysis Software automatically adds the ladder using the signal from Bio-Rad Precision-Plus Protein Marker (M kDa).

*Digital Processing of Raw Gels*:

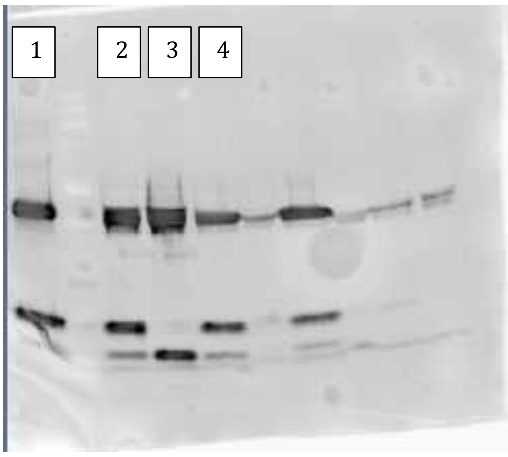


The figure above is the digital-scanning of a blot of the acrylamide gel onto nitrocellulose membrane. The raw image for SPF 2-C above is the first digital scan that the Bio-Rad Pharos FX$^{TM}$ Plus Molecular Imager does (shown at the left of this text). The ladder is also blotted into the nitrocellulose, Bio-Rad ladders for the Bio-Rad Pharos FX$^{TM}$ Pharos Scanner system were used. The order in the first scan of the raw gel (left) is [sample]-[ladder]-[8 x samples].
BioRad Quantity-One 1-D$^{TM}$ and PDQuest$^{TM}$ 2-D gel analysis software were used to capture and to reorganise the first digital scan to show it in the order of SP2-C. The Bio-Rad gel digital-analysis platform and software tools allow the editing (i.e. moving lanes) **BUT always preserving the gel alignment**. The Bio-Rad Pharos FX$^{TM}$ Pharos scanner and software create gel anchors that allow quantification, warping, image processing and editing while preserving the all gel-data spatial integrity. All protein gel data and their final figures as SP2-C above, were analyzed and produced using this Bio-Rad system with the lanes shown in SP2-C above being from the same raw gel shown here. The only post-editing of the figure outside of the Bio-Rad software was to label wells and making ladder lines clearer for the final figure SP2-C included in the supplemental material.

**Supplementary Figure 4**.

Linear regression age vs. plasma Haptoglobin in the malaria-negative Community Control group showing that plasma haptoglobin levels are independent of age.

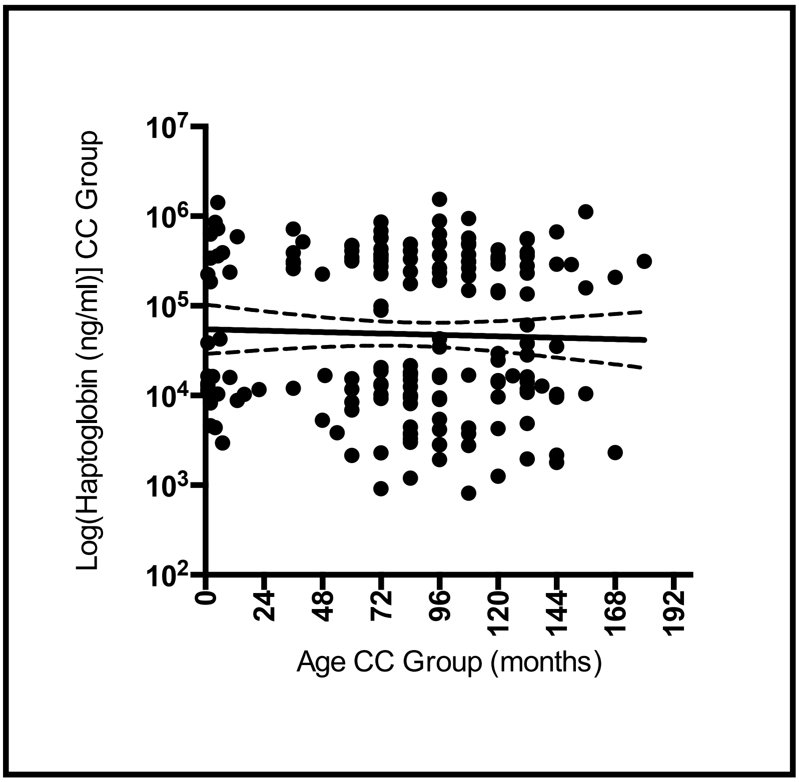


Linear regression: 95% Confidence intervals. $R^2$=0.001129; F=0.1921; p=0.6617; N=174. Solid line (mean) dotted line (95% CI)
